# Brave New World‡

**DOI:** 10.4269/ajtmh.22-0447

**Published:** 2022-08-22

**Authors:** Julie Jacobson

**Affiliations:** Bridges to Development, Vashon, Washington

4,919,755. What’s in a number? Well, we know that’s a big number, but it’s also abstract. What’s in a number? I would say everything, when your loved one is reflected in that figure of 4,919,755 deaths due to COVID-19 as of October 2021.^1^ How do we convey the weight of this number? We are all impacted by this global situation. We’ve all had loss—lost health, loss of a loved one or colleague, economic hardship, extreme stress. We’ve all suffered. And at the same time, we’ve been held away from each other by social distancing and masks, missing our ways as human beings to replenish and comfort each other by not being able to share physical contact and connection.

The pandemic has made us, forced us, to question all of our assumptions. Who are we as a people? How are we doing in this greatest challenge of our time? We’re all sharing in the same global event with all our own unique perspectives and experiences. How have you grown? Changed? Where will you be when all of this is over and we emerge on the other side? Will we have a Brave New World? Will sharing a global calamity provide an opportunity to bring us together in a way that nothing has before? The American Society of Tropical Medicine and Hygiene (ASTMH) themes this year—“Compassion, Culture, and Courage”—are intended to be touchstones in this journey to our Brave New World.

What’s in a name? Brave New World:

Oh, wonder! How many goodly creatures are there here! How beauteous humankind is! Oh, how Brave New World that has such people in it.^2^

This is Shakespeare, and I actually do believe humankind is beauteous when I look out at the ASTMH family and all the amazing, talented minds dedicated to making the world better, safer, healthier. In the case of this quote, however, the speaker is misguided and not understanding the nature of the scene she’s seeing. And it’s this misunderstanding that inspired the title of the book, the science fiction book by Aldous Huxley^3^ in the early 1930s. The book shows a fear that’s pervasive today and still relevant—a dystopian future society, science run amuck. Babies cultivated in incubators, predetermined to fill certain roles, everything controlled, no freewill.

This is what people fear in science: the robots taking over, humans being enslaved, changes determined by science, and things and people they don’t understand or trust. And today this is our challenge. The fear of science and misinformation that has now become commonplace is the biggest threat to our health and security. I want to remind you of a quote I heard from Bill Foege during our fireside chat, “Science does not have a moral compass.”^4^ That’s where we come in. That’s our job. Put the science to good use in service of the people. So, how do we communicate? How do we communicate what we do? Where do we turn? Where can we learn? How do we share that we are all in this together?

I want to share with you some inspiration and some ideas that I’ve come across this past year or so that I think have things to teach us. They come from some unusual sources: two lawyers, a car salesman, a Swedish physician, and a spy.

The Sum of Us. One thing the pandemic has taught us is that we’re all connected. For some, this brings up deep fears. It has inspired nationalism and racism and violence. I, however, want to share with you a beautiful thinker who offers us another option, Heather McGhee. She’s a lawyer. She’s also a self-described economic policy geek. And she has a new book, *The Sum of Us*,^5^ S-U-M, and it’s brilliant. It introduces the concept of the “solidarity dividend.” This challenges a concept that’s very pervasive: the zero-sum game, that there is a winner and a loser in all interactions. And if I win, you lose, and if you win, I lose. This means I don’t want you to win because that would somehow imply that I lose. This has led to policies and practices that hurt everyone.

Institutional racism plays to this zero-sum game thinking. If I do something that helps you somehow, I will lose. However, that thinking brings down everyone in the process. The idea of the solidarity dividend is that we all actually do better when we work together. Heather explores the policies that have been the underpinnings of systemic racism, which many of us have inadvertently participated in without our knowledge or understanding, and how those policies have hurt us all economically and socially. Most importantly, she gives us hope that through understanding and shining a light on these policies and how they have hurt us, we can change, and achieve more, and receive the solidarity dividend.

She digs deeply into policies and U.S. culture, demonstrating a deep and persistent courage through this exploration to look and learn and show us our shared history that’s been hidden from us. Only by understanding this history can we create a new path to a Brave New World.

For me, this analysis is very helpful, not only for understanding U.S. policy, but in understanding some of the legacies of colonialism and how they similarly may hold us back in our work, in our progress in tropical medicine and global health.

The second concept I think we need to bring us into our Brave New World comes from another lawyer, Valarie Kaur. She’s a social activist. The lesson I have received from her is the inherent danger in the “us and them” mentality. She offers a constructive approach to move past that in her book *See No Stranger*.^6^ It actually fits quite nicely with the work of Heather McGee, who thinks in systems and policies, whereas Valarie Kaur thinks of individuals and makes it very personal. She puts power into the decisions and the actions we take every day, understanding people that we think of as opponents as others, instead of as people like ourselves, striving to know and understand how they came to be in apparent opposition to us.

This approach to understanding will be very helpful to us as we try to understand anti-science, anti-intellectual, and anti-vaccine thinking. Can we address the underlying issues and find constructive ways to collaborate and communicate? She has a very good TED Talk if you would like to learn more.^7^ She offers a very interesting “compass” with which to start to address and reach out to people we feel in opposition to or in conflict with. It’s also on the website, the Revolutionary Love Project (https://valariekaur.com/revolutionary-love-project/). In her work, she demonstrates finding compassion for those who have hurt you, and understanding what has led them to be who they are—encouraging and finding ways to communicate with people who think and act differently to find a new and more constructive path forward.

Another great thinker of our time, Hans Rosling, who has sadly passed, also fought the concept of “us and them,” “the West and the rest,” “the developing world and the developed world.” His quest to understand this dichotomy and how a misconception of this dichotomy drives poor decisions in planning and suffering led to a unique life and legacy. Founding a project called GAPMINDER with his son, he challenges us 1) to find your misconceptions, 2) to understand a changing world, and 3) to see the reality behind the data. If you have not seen his TED talk^8^ or been to the GAPMINDER website (https://www.gapminder.org), you are really missing something special and enlightening. His visualizations of data and his drive to communicate the data meaningfully are inspirational.

**Figure 1.**
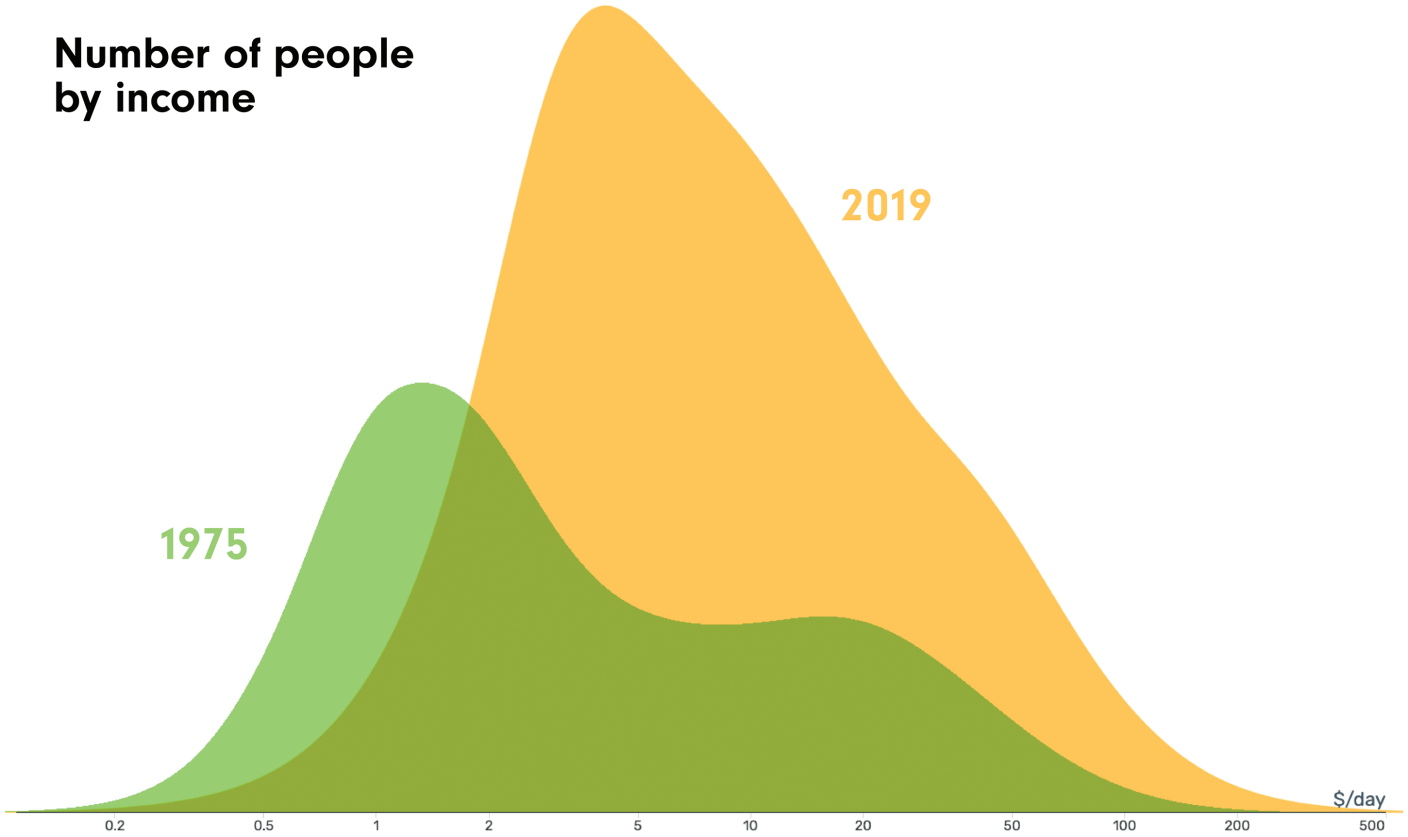
This graph shows the number of people by income in the world in 1975 and 2019. In 1975, the distribution of wealth was in two mountains, showing a dichotomy between the poor and the rich, whereas in 2019, the distribution of wealth is now in one mountain, with most people in the middle.^9^

His message in the work of GAPMINDER is to show us that we all have big misconceptions about the world. These misconceptions are driven by our upbringing, our education, and the news. The first of these misconceptions he tackled was the concept of “us and them,” which is illustrated in Figure 1.^9^

This dichotomy was based on the concept of this two-hump graph—very poor on one end and rich on the other. However, I want to point out that these data are from 1975. When we look at more recent data, we see one hump; we have a world that’s moved to the middle, with the extremes less defined, and this shift has changed the nature of the problems we were trying to solve, and who is there to solve them. We need to understand these changes and what they mean for our work.

I next move to the car salesman, Alan Mulally. Actually, he was the chief executive officer (CEO) of Ford, but he sells cars. They were having a bad year—lost $5 to $6 billion and projected to lose up to $17 billion. Alan called his managers together to look at progress, challenges, and to rank issues that they were having: red, bad; yellow, concerning; green, good. The managers all came together. Of course, they’re with their big boss, the CEO. No one had any red on their slides. This, of course, caught Alan’s attention. How can we have lost $6 billion and have had no problems?^10^

This started a discussion and a debate, and soon the managers were sharing that there actually was quite a bit of red, but nobody wanted to show weakness or admit failure. However, it was only by opening up, and by bringing the problems and sharing solutions across teams and locations that things started to change. And only by looking at where there was trouble, sharing lessons learned, and brainstorming new approaches was the company able to turn itself around and get back on the road to success. This example, although we are not selling cars, but instead, trying to save lives, shows us it’s important to reflect openly on our challenges and course-correct for the future, acknowledging what is not working.

Okay. I went to see the recent James Bond movie, *No Time to Die*. It was my first movie in 2 years due to the pandemic. It was kind of exciting. He always has cool toys, mostly that blow things up or help him get away quickly. But this time the plot included, and I don’t think I’m ruining anything here, DNA-encoded nanobots. Of course, they caused lots of trouble. But I found myself reflecting on this after the film, and realize that in tropical medicine, we already have these. So, what if we had 00ASTMH instead of 007? Let us consider *Onchocerca volvulus*, the cause of onchocerciasis. This crafty parasite comes in unknown through the bite of a fly, matures, and reproduces without the host even knowing that it’s there, living in the skin and causing uncontrollable itching. And you can’t wash it off. You can’t get it off, because it’s in your skin. Eventually, it gets into the eyes and slowly causes blindness. That one’s pretty good.

Chagas. This one’s even better. The kissing bug bites you in your sleep. And you inoculate yourself with the bug’s feces by scratching the wound. It gets into the body, hides in the smooth muscle, destroying your heart, destroying its ability to beat or your colon’s ability to excrete. Sounds pretty terrible.

Human African trypanosomiasis, the bite of another fly. This one lives in your body, multiplying and finding a way to break through the blood–brain barrier into the brain, where it disrupts your sleep patterns until you slowly go crazy and die.

What about the high-speed chase of malaria in your bloodstream? Changing costumes, it hides in the liver and then emerges, infecting your blood cells and bursting them from within.

Schistosomiasis. You innocently contact a body of water—maybe getting a drink; washing your clothes, yourself; cooling off on a hot day. Little do you know that just by contact with that water, you’ve been infected with a deadly parasite that can kill your liver; destroy your bladder; embed eggs throughout your genital tract, causing bleeding, pain, and—for women—increasing your risk of acquiring HIV and threatening your ability to have a baby.

Our stories are exciting, and our parasites are tough and scary. We have fewer tuxedos and martinis, but our world is no less exciting. International intrigue, overcoming adversity, global partnerships, millions of people’s lives at stake. We need to tell our stories and have the world rooting us on to be victorious. How many will see this lecture? Okay, we have our dedicated members of ASTMH and all of the attendees—4,000 to 5,000 max—but we’re not at James Bond levels yet. We have a gap. So, what does this mean for ASTMH? It’s now time for us to look at ourselves, at our work, our colleagues, our institutions, and determine how we can overcome our limitations, challenge our assumptions, see our bias, and rebuild our systems in service of our mission in a Brave New World.

Why does it take 20 years to develop a new treatment of a neglected tropical disease? Is that acceptable? Why do we accept that? Why does it take 20 years to get a lifesaving vaccine that’s used in a rich country into a country with less means? Can we create a system that looks at need, and not resources, as the driver for action? We have a lot more to learn from each other. Bench scientists, entomologists, clinicians, social scientists, advocates; we’re all part of the solution and we all have a piece of the puzzle.

ASTMH’s mission, dedicated to reducing the worldwide burden of tropical infectious diseases and improving global health; generating, sharing scientific evidence; informing health policies; fostering career development; recognizing excellence; advocating for investment in tropical medicine. This inspires me to more “C” words beyond compassion, culture, and courage: communication, community, collaboration, and creativity. These are all inherent in our model, and we need to continue to nurture them. So, let’s let the data guide us.

**Figure 2.**
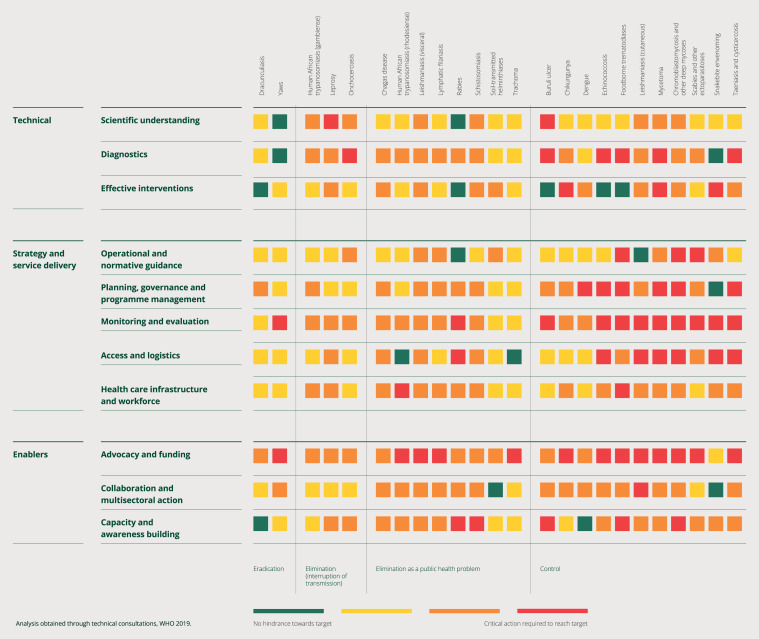
Heat map for the gap assessment for each neglected tropical disease.^11^

We need to see where we have problems, to see where we can work together to solve them. The gap assessment done for the 20 disease areas in the WHO neglected tropical disease (NTD) global program shows that we’re not afraid of red, although, perhaps we should be (Figure 2).^11^ We see from this gap assessment that there is a lot of work to do, and if we look at the areas that are highlighted, they range from diagnostic tools to partnerships and advocacy. We see that we need many skills and cross-sector collaborations to make this work successful and to turn some of these reds into greens.

Malaria and NTDs have similar scorecard exercises with the African Leaders Malaria Alliance. These exercises look at national programs and their needs and challenges. Only by following progress and looking at the challenges ahead will we change the status quo. We are one step ahead of Ford in understanding our challenges, questioning our assumptions.

**Figure 3.**
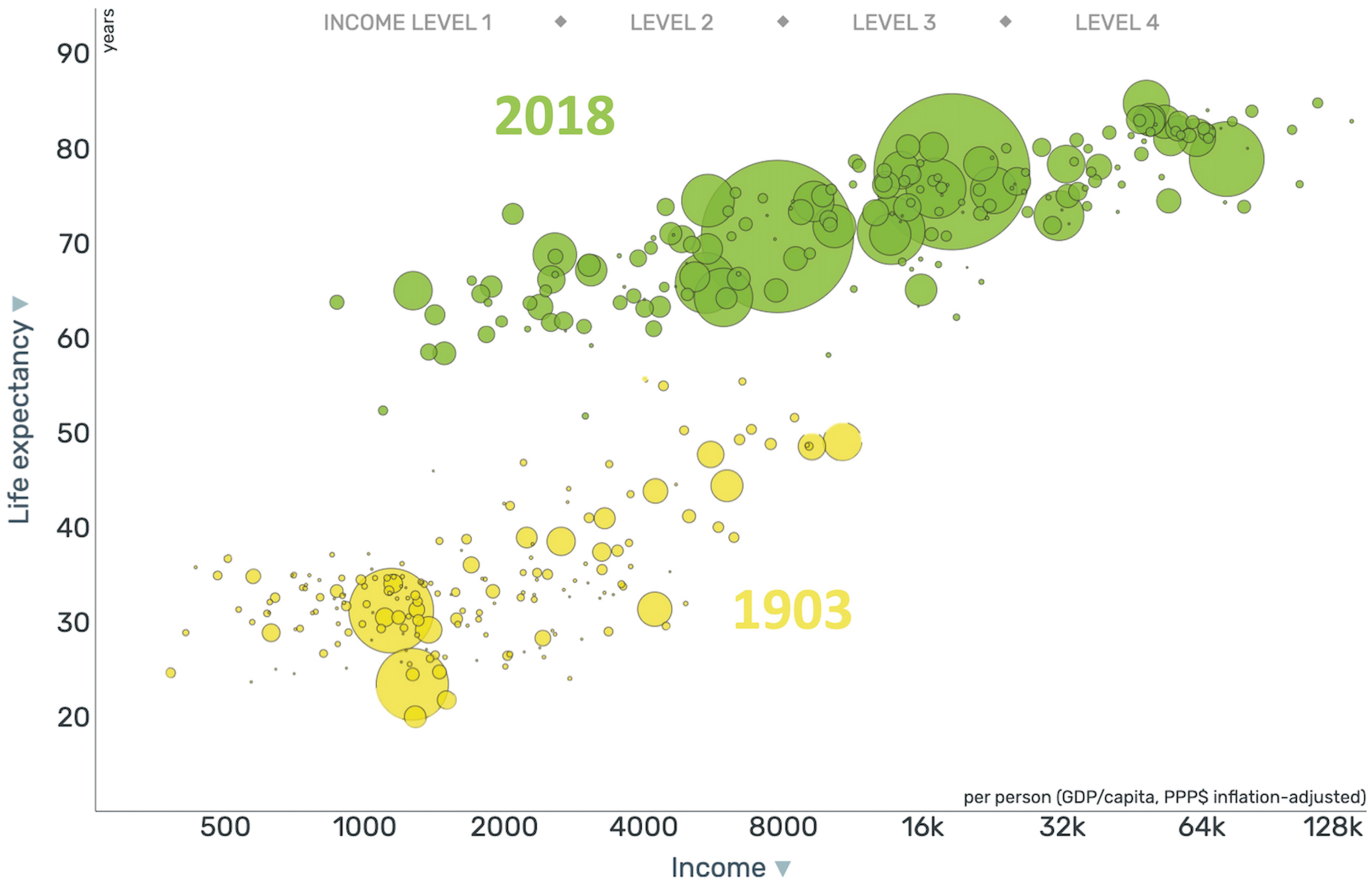
Graph showing life expectancy and income by country (each bubble is a country) in 1903 (yellow) and 2018 (green). As both have increased, this shows that the quality of life is improving.^12^

One thing that it’s easy to lose sight of right now, with the challenges of the pandemic and all the subsequent issues impacting us acutely, is if you step back and look at the data, the world is getting better. In the work of Hans Rosling visualized in GAPMINDER graphs, over the past 100 years, life expectancy and income by country have both increased around the world, indicating that quality of life is improving (Figure 3).^12^

**Figure 4.**
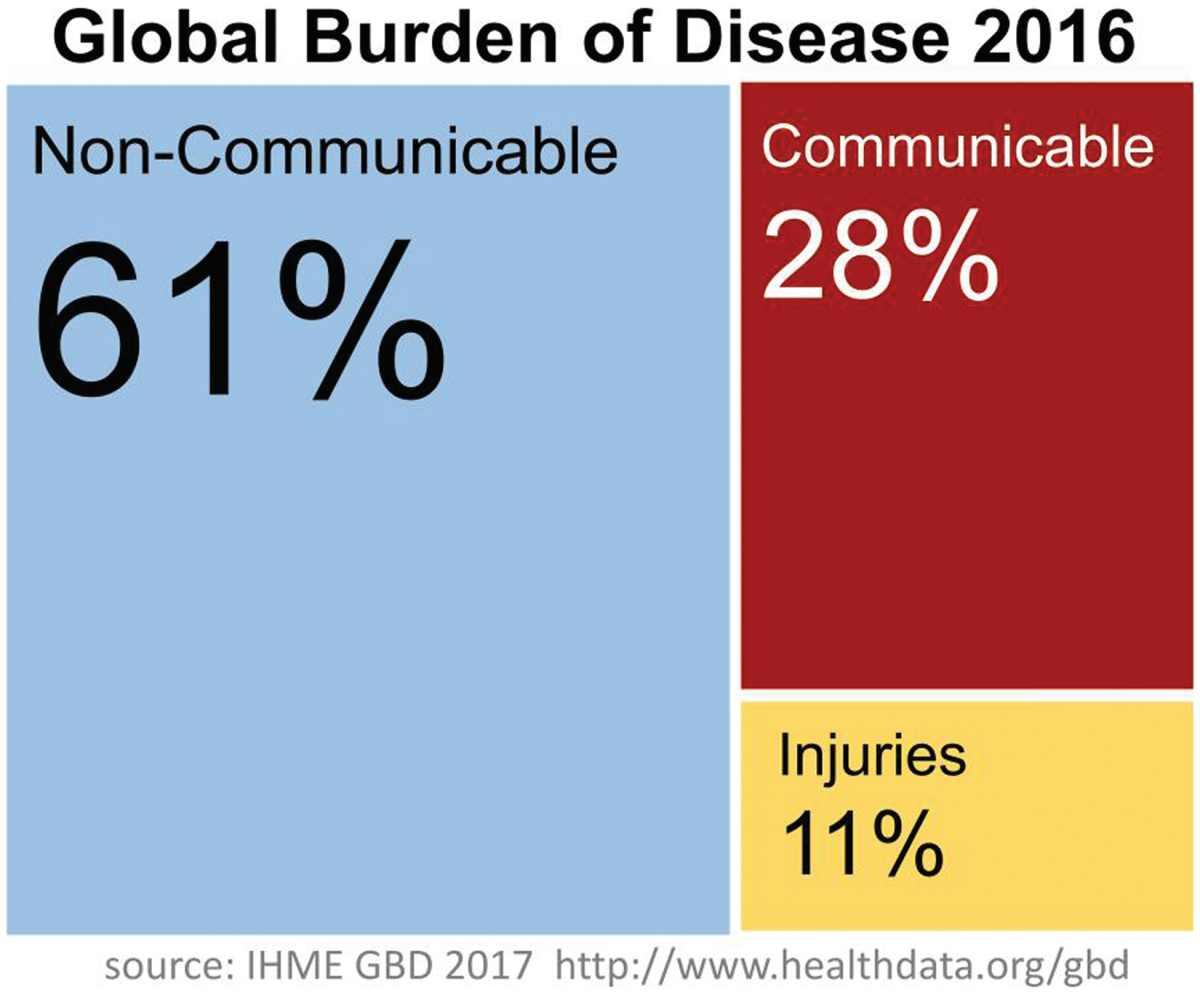
Graph showing the distribution of the global burden of disease by communicable, non-communicable, and injuries.^15^

**Figure 5.**
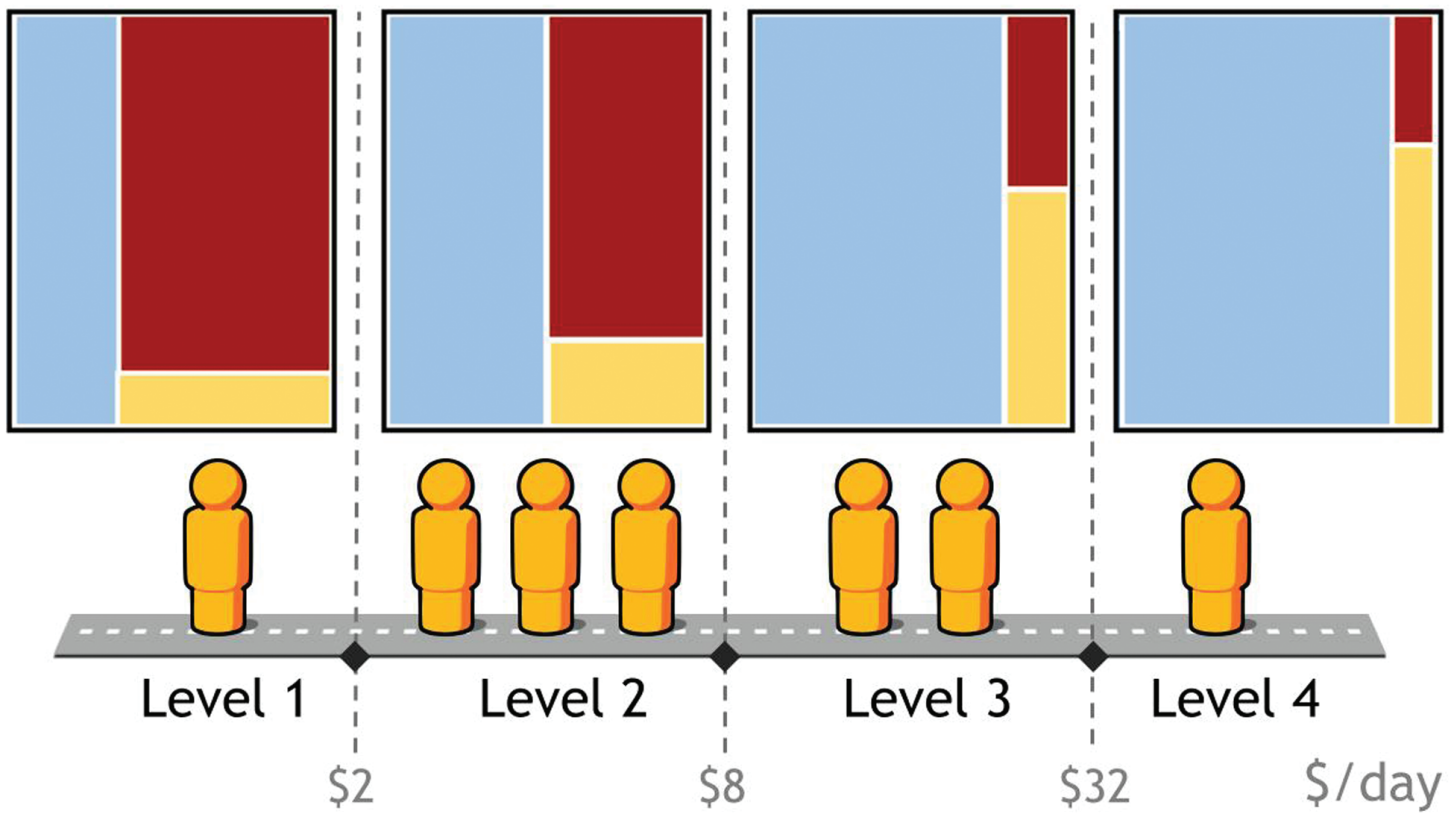
Each of these figures, represents a billion people, so this table shows how many billion live in each of these income ranges and how the proportion of global disease burden changes in each.^15^

Since ASTMH was founded in 1903, there has been a lot of progress. People are earning more money and living longer, and that’s good news. Women are having fewer babies, and more of the babies are living.^13^ Extreme poverty continues to decline.^14^ The burden of disease has changed. Data from 2016 from the Institute for Health Metrics and Evaluation shows the global burden of communicable disease, non-communicable disease, and injuries (Figure 4).^15^ These proportions have shifted significantly as global progress has been made. However, this shift is not universal, and the data show us that the proportion of communicable diseases increases with decreasing wealth. Each of these figures, each of these little people, represents a billion people, so you can have an idea of how many people live in each of these income ranges and how the proportions change (Figure 5).^15^

The importance of our work to the billion being left behind is profound, but we also need to keep an eye to the future and the challenges ahead. These changes will need to guide our work as a society to be sure that we do not forget our mandate and leave that 1 billion behind who remain, with communicable diseases holding them back, or ignore the new challenges that lie ahead.

I want to share with you an area where my own assumptions were questioned and challenged in the past year: female genital schistosomiasis (FGS). The situation as I saw it: no one cares. No one knows about FGS. It’s one of the best kept secrets. It’s not in medical education. I know that it’s not seen in textbooks. It’s invisible, except for those people that it affects. It has no disability adjusted life years (DALYs), so it doesn’t exist in priority-setting. It isn’t reported, so it’s not prioritized in health systems, and it generates no record in the health system and therefore no DALYs. It becomes a self-fulfilling prophecy in a perfect storm of neglect.

People who work in sexual and reproductive health (SRHR) don’t know about it. People who work in SRHR programs never suspect a parasite when a patient presents. The symptoms mimic sexually transmitted infections with vaginal discharge, pain, and bleeding. Eggs from the parasite embedded in the genital tissue cause granulomatous inflammatory responses that lead to ectopic pregnancies and are associated with a 3- to 4-fold increased risk of acquiring HIV. All of this for the want of praziquantel, an inexpensive treatment—often even free—as the drug is donated by the Global Health Institute of Merck KGaA.

I will share two stories in the past year that challenged my assumptions with regards to FGS. We had some funding from Canadian Grand Challenges from the Canadian government with matching funds from many partners. The full project is called the FGS Accelerated Scale Together, or the FAST Package. There will be a talk here at ASTMH on the whole project, if you would like to learn more.^16^ In the work I will discuss, the funding came from the Merck Global Health Institute and from the Task Force for Global Health as part of the matched resources.

First, we needed competencies. What do people need to know at each level of the health system? Our first approach was to do this the way we always do this: a WHO meeting. We gather a handful of global experts, a couple of people from endemic countries that we have enough money to send to Geneva. Then COVID happened, and there was a global standstill. Now what do we do? We need to find a new way. So, we worked online, and through this process we learned a lot. We started by inviting the principal investigators of the projects that had been funded recently by the Task Force for Global Health Coalition for Operational Research on NTDs, or COR-NTD. From this small group, word got out, and more people requested to be part of this workshop where we collaboratively established competencies for working practitioners in both health facilities and in communities.

We ended up with an amazingly diverse online group, with 65 participants from 24 countries. The online format allowed us to work together to hold small-group discussions, then work on what we had been provided that day, analyzing and cleaning it, and bringing it back to the group for further discussion. We worked over 3 weeks and we ended up with a much higher quality product than we would have ever had after a short in-person meeting. And there is now a paper on the development of the competencies that’s being published in the *British Medical Journal of Sexual and Reproductive Health*.^17^

The second time my assumptions were challenged was when we took these competencies and put them into a training program. We put out a notice online that this training was going to be available in English and French, and we included those who helped identify the competencies in the invite. Then, 1,527 health practitioners took the time to apply. We were shocked. Applicants came from many different levels of the health system and from many countries. We conducted the course in English and French over a 3-week period. And, unfortunately, we could not train everyone. We could only take 20% of those who were interested, but the response showed that people did care, and they did want to know, and there was a huge demand and an interest in an area where we never suspected it, but hoped that it would be.

The recent progress in NTDs has been born in questioning assumptions. In 2012, the WHO published the 2020 road map for NTDs. This could have been just another document giving ambitious goals with little progress, but it wasn’t. There was a response from the collective set of partners who were committed and required to commit something to achieve the progress that was set out in those 2020 goals, which was codified in the London Declaration.^18^

**Figure 6.**
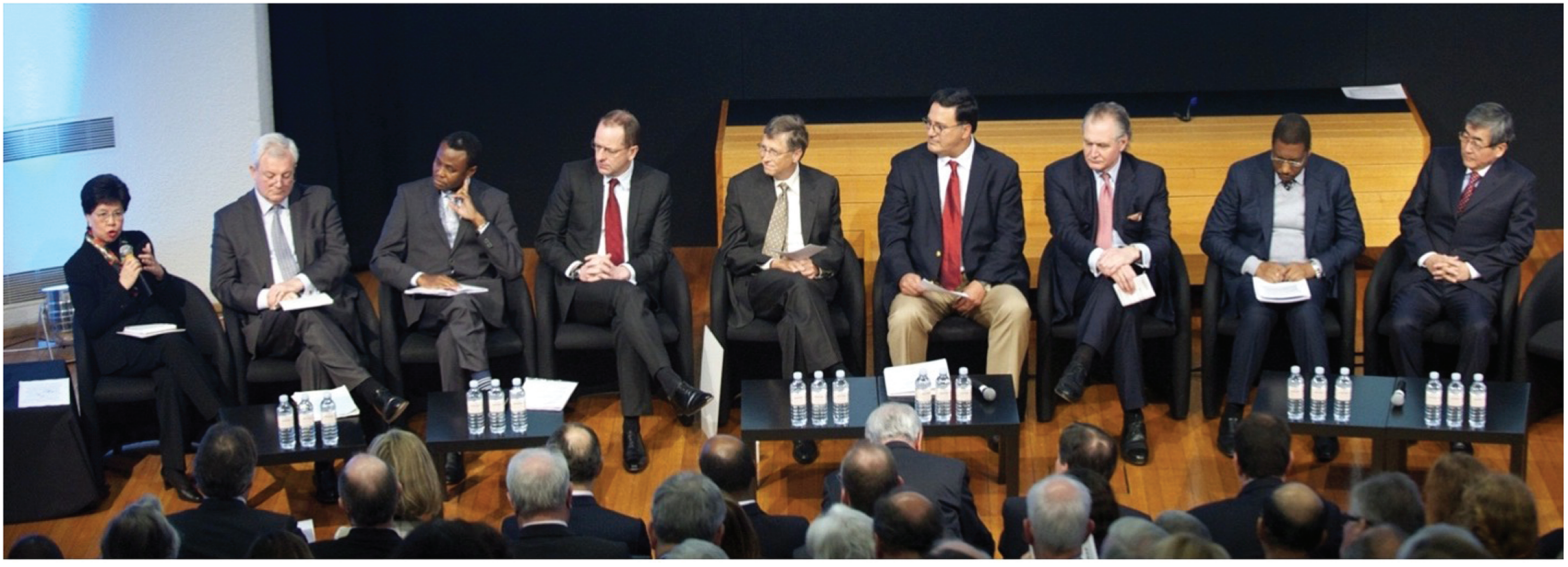
Signatories at the launch of the London Declaration on Neglected Tropical Diseases, 2012.

This was a simple document, basically saying, “I will do my part.” And it started the partnership of Uniting to Combat NTDs. In this picture, you see nine competitor CEOs sitting with two Ministers of Health and the Director General of the WHO, all aligned to do their part (Figure 6). And now, almost 10 years later, we see such progress. Since 2015, more than 1 billion people have been reached annually with preventive chemotherapy for NTDs. We have a *Guinness Book of World Records* entry for the largest pharmaceutical donation ever in the history of the world.^19^ And 43 countries have eliminated at least one NTD by 2022.^20^

ASTMH is a part of this story. It was at ASTMH, the conversations over coffee or a meal, a quick catch-up in the hallway, that made the building blocks for the London Declaration and Uniting to Combat NTDs, where these challenges were discussed and solutions were found.

There are many amazing accomplishments to celebrate (see Table 1). And, actually, at the top of this list is the malaria vaccine now recommended by the WHO. Many years of work and dedication made this accomplishment come to light. That’s true for everything on this list. New oral treatment of human African trypanosomiasis with fexinidazole; the triple-drug therapy for lymphatic filariasis; moxidectin, the first new drug for onchocerciasis; benznidazole for Chagas; and post-exposure prophylaxis for leprosy that actually decreases the incidence of new cases.

**Table 1:** A sample of recent accomplishments in tropical medicine and hygiene

Amazing major accomplishments to celebrate
First-ever parasite vaccine for malaria now recommended by the WHO New oral treatment of human African trypanosomiasis: fexinidazole Triple-drug therapy with ivermectin, diethylcarbamazine, and albendazole for accelerating the elimination of lymphatic filariasis Moxidectin: the first new drug for onchocerciasis Benznidazole available for the treatment of Chagas disease and licensed in the United States Post-exposure prophylaxis for leprosy to decrease the incidence of new cases

The other thing that these accomplishments have in common is that ASTMH was part of the story. The science, the discoveries, the data were discussed and debated here, and they benefited from those hallway conversations, the chance meeting, the opportunity to share and collaborate that the Society provides. And that’s part of what makes the Society special. All of you and your work that will be shared over the next few days, years—and the ongoing collaborations—will continue to bear fruit.

The most amazing development is the development and deployment of a vaccine for COVID. I want to remind you: on March 11, 2020 the WHO declared COVID-19 a global pandemic. In December, the first vaccine from Pfizer was authorized for emergency use, quickly followed by Moderna, J&J, Sinopharm, and others around the world. By mid-October 2021, 6.8 billion doses had been administered. That is amazing. Now, was it equitably distributed? No, but this is nonetheless an unbelievable accomplishment: 48% of the world has had at least one dose of the vaccine.

We know, however, that coverage is only 3% in low-income countries. Time is cycling faster than ever, and I feel we judge ourselves too harshly. COVID has created a global experiment to bring equity and access to vaccines. When we think about access to lifesaving vaccines, like hepatitis B or the one that I worked on for many years, Japanese encephalitis, these vaccines were not available to the populations who needed them most for more than 20 years after approval. Here, with COVID, we may have successfully had a vaccine available worldwide in record-breaking time. Less than 1 year after the vaccine first became available, it is used in at least 34 countries.

The most important thing is that we’re having the debate. We’re discussing, we’re arguing, we’re fighting for equity. A global voice has been raised to a global challenge. This is a new conversation. It’s a shared experience. It’s brought us together. COVAX and its successes are happening now. Should we be outraged and demand more? Yes, of course. The limitations in our global infrastructure, the need to invest in health, so much work to be done. But I also think we should be proud, proud that we have come so far as a people and a planet. We should take a moment in the midst of all the action, the overtime, to breathe and realize we’ve come a long way.

So, some closing thoughts. We need to understand our history. We cannot let this challenge divide us. We must find a solidarity dividend. We must grow stronger and more resilient as a human race. We need to rebuild trust, and only by understanding each other and caring for each other can we find a place where trust can come forward again. We must strive to find ourselves in others and see no strangers. The world is different. We are evolving. Things are getting better, and you can be part of that change. We need to tell our stories. We are in a new chapter in history. We still have real challenges ahead, but together we are stronger. It will take us all to apply our talents, use the data, question the assumptions and the status quo.

So, what does a Brave New World mean to you? A world without malaria? A world without Guinea worm? A world where everyone’s contribution is valued and counts? A world where parasites don’t determine your fate because of where you were born? What needs to continue, what needs to change, what needs to evolve, one step, one study, one piece, one handshake, one collaboration, one smile, one hallway conversation at a time? A world where opportunity is given to the best ideas regardless of who you work for, your country of origin, how much money your family made, how much money you made, what kind of house you lived in, what God you believe in, or who you find attractive? We need to find a way to nurture humankind, find solutions and overcome adversity, and to find balance and joy.

You remember joy? It’s still there. This is not over, whether it’s the struggle to get vaccines, the threat of the evolving virus, the lingering economic hardships or psychological challenges that have come from isolation, or the loss of a loved one. These challenges remain with us and we will have to see how we can overcome them, because we will. And what kind of world will we build together in the process? Over the next few days, take the opportunity. It’s harder in the virtual format, but all the more important, to acknowledge a colleague or a friend for demonstrating one of the three Cs: showing compassion, honoring culture, demonstrating courage. Let’s nurture and support one another and see things the way we want to move forward into our Brave New World.

Thank you.
